# The role of quorum sensing in rhizosphere community regulation during bacterial wilt pathogen invasion

**DOI:** 10.3389/fpls.2026.1685007

**Published:** 2026-02-16

**Authors:** Bin Zhang, Yanyu Liu, Deying Zhou, Yuangang Lv, Mingfeng Cao, Hengli Li, Zhaoyue Yang, Zhenghua Liu, Huaqun Yin, Xichun Wang, Zhihua Huang, Delong Meng

**Affiliations:** 1 College of Chemistry and Bioengineering, Hunan University of Science and Engineering, Yongzhou, Hunan, China; 2 School of Minerals Processing and Bioengineering, Central South University, Changsha, China; 3 Yongzhou Company of Hunan Tobacco Company, Yongzhou, China; 4 Yongzhou Municipal Bureau of Agriculture and Rural Affairs, Yongzhou, China; 5 Changde Tobacco Company of Hunan Province, Changde, China; 6 Yuxi Company of Yunnan Tobacco Corporation, Yuxi, China

**Keywords:** bacterial wilt, metagenomic, microbial communication, quorum sensing, rhizosphere microbiome

## Abstract

Bacterial wilt, caused by the soil-borne pathogen *Ralstonia solanacearum* is a major threat to solanaceous crops worldwide. The onset of this disease is frequently associated with disruptions in the rhizosphere microbial community. Quorum sensing (QS), a key mechanism for microbial communication, plays a critical role in regulating microbial interactions and maintaining community structure. However, whether and how QS is involved in reshaping the rhizosphere microbiome during *R. Solanacearum* infection remains poorly understood. In this study we compared QS-related genes, signaling pathways, and network structures in metagenomes of healthy and wilt-infected rhizospheres. The results show QS-related genes of the plant beneficial bacterial were significantly down-regulate, whereas QS-related genes of pathogenic *R. Solanacearum* were up-regulated in wilt-infected rhizosphere. The up-regulated QS genes of pathogens belong to eight QS signaling pathways (AI-1, GABA, PapR, NprX, Phr, cCF10, and DSF). Network analysis showed a simplified structure in the wilt-infected rhizosphere. It is also found the number of connectors in the QS gene co-occurrence network was reduced in wilt-infected rhizosphere network. This is due to the upregulation of QS system allows the pathogen to mediate the rhizosphere microbial ecology network, and leads to destabilization of rhizosphere community. These findings demonstrate that QS system contributes to bacterial wilt infection by suppressing the QS-based interactions among plant beneficial microbes, thereby triggering community function disruption.

## 1 Introduction

Bacterial wilt, caused by *Ralstonia solanacearum*, is a destructive soil-borne disease prevalent in tropical and subtropical regions. The pathogen invades the plant vascular system, disrupting water and nutrient transport, leading to rapid wilting and eventual death. It poses a serious threat to the yield and security of many important crop (Hayward, 1991). In the rhizosphere, the structure and function of microbial communities are key determinants of plant health and disease susceptibility (Ali et al., 2021a). At a broader ecological scale, soil biogeochemical and stoichiometric constraints have been shown to synchronously regulate ecosystem nutrient dynamics and biological processes, thereby indirectly shaping microbial community assembly and function (Liu et al., 2023). Increasing evidence shows that rhizosphere microbes not only contribute to plant nutrient cycling and hormone regulation, but also help suppress pathogens through nutrient competition (Gu et al., 2020; Trivedi et al., 2020), production of antimicrobial metabolites (Zhou et al., 2022), and induction of systemic resistance (ISR) (Salwan et al., 2023). The structural complexity and functional stability of rhizosphere communities are considered critical ecological factors for plant defense against soil-borne diseases. Our previous work showed that healthy plants in bacterial wilt-affected soils tend to harbor more complex microbial interaction networks in the rhizosphere (Zhao et al., 2025). Other studies have also reported that highly connected microbial networks can stabilize community function through cooperative metabolism and signal exchange, limiting pathogen invasion and colonization (Fakruddin et al., 2023; Ge et al., 2023; Liao, 2025). In contrast, simplified microbial communities are more unstable and vulnerable to pathogen intrusion.

Quorum sensing (QS) is a well-studied mechanism of microbial communication that can regulate microbial group behavior in the rhizosphere. Through the production and detection of small signaling molecules (such as AHLs and AI-2), QS enables microbes to sense population density and coordinate collective behaviors, including biofilm formation, extracellular enzyme secretion, secondary metabolite production, and chemotaxis (Abubakar et al., 2023; Bridges et al., 2022; Cella et al., 2023; Li et al., 2025; Sharma et al., 2020). This system helps microbes adapt to environmental stress and plays a central role in shaping community structure (Liu et al., 2022). In practical agricultural management, the detection of relevant microbial signaling molecules facilitates sensitive and rapid detection and identification of plant pathogens, thereby effectively preventing the occurrence of plant diseases (Ali et al., 2021b, 2022; Tang et al., 2025). In addition to QS-mediated coordination, quorum quenching (QQ), defined as the disruption or degradation of QS signals, represents an important counter-regulatory mechanism in the rhizosphere (Grandclément et al., 2015). Numerous beneficial bacteria, particularly well-studied Bacillus species, are capable of suppressing QS in plant pathogens by producing AHL-degrading enzymes, such as lactonases and acylases (Bzdrenga et al., 2017), as well as through other QQ mechanisms (Das et al., 2022), and effectively attenuate pathogen virulence and biofilm formation (Paluch et al., 2020; Rosier et al., 2021; Xu et al., 2024). In practical agricultural management, the development of environmentally friendly plant disease suppressants is of critical importance (Ali et al., 2022a). These QQ-based strategies disrupt pathogen communication systems without exerting strong selective pressure for resistance development in non-target microorganisms (Ahmed et al., 2024; Deng et al., 2014; Dong et al., 2000), are widely regarded as ecologically sustainable approaches for biological disease control.

Rhizosphere bacteria can inhibit pathogens by regulating antimicrobial compound production via QS, or by disrupting pathogen QS signaling through QQ (Ahmed et al., 2024; Rodríguez et al., 2020). Gu et al. further reported that some “pathogen-aiding” strains in the rhizosphere may promote *R. Solanacearum* growth. Disrupting the interactions between these strains and the broader microbial community can enhance suppression of the pathogen (Li et al., 2022). Previous studies have demonstrated that QS systems are involved in modulating disease development. *R. Solanacearum* regulates virulence expression through QS pathways such as AI-1 and VFM, and disruption of these pathways significantly inhibits disease progression (Yoshihara et al., 2020). Pseudomonas aeruginosa maintains virulence levels by modulating rhlI activity and recalibrating the concentration of signal molecules to stabilize RhlR-mediated QS signaling (Simanek et al., 2023). Most plant pathogens share highly conserved QS regulatory mechanisms, which play essential roles during pathogenesis (Baltenneck et al., 2021). Collectively, Quorum Sensing (QS) represents a central communication framework for microbial coordination; it is indispensable not only for the fine-tuning of pathogenicity but also as a key mediator of rhizosphere homeostasis and integrated disease management.

Although quorum sensing (QS) systems regulating virulence in *R. Solanacearum*, such as AI-1 and VFM, have been extensively characterized at the single-pathogen level, previous studies have primarily focused on pathogen-intrinsic traits, including virulence factor expression, motility, and biofilm formation. Consequently, far less is known about how QS-mediated interspecies communication operates at the community level in the rhizosphere. In particular, it remains unclear how pathogen-centered QS signaling reshapes microbial interaction networks and disrupts cooperative functions; and the reorganization of QS-related functions across the entire rhizosphere microbiome during bacterial wilt progression has not yet been systematically examined, particularly regarding how shifts in QS network architecture influence microbial connectivity and functional stability.

To address this question, we investigated rhizosphere soils from healthy and diseased plants sampled within fields where bacterial wilt was present, using metagenomic sequencing to systematically compare the composition, structure, and metabolic functions of their microbial communities. We focused on QS related functions, analyzing differences in QS gene abundance, signaling pathway architecture, and network connectivity. Our results show that *R. Solanacearum* gains a central role in the community QS system, allowing it to interfere with and reshape rhizosphere QS communication. This disruption drives a shift from a multicentric, cooperative, and stable microbial network to a unipolar and destabilized structure centered on the pathogen, creating conditions that favor disease development. This work provides new insights into how QS mediates the transition of rhizosphere communities from health to disease and suggests potential targets for signal-based disease control.

## 2 Methods

### 2.1 Sample collection

Rhizosphere soil samples were collected from a tobacco field in Guiyang County, Chenzhou City, Hunan Province, China (25°75′97″N, 112°74′05″E), a region with a subtropical monsoon humid climate where tobacco is a major crop. Under natural field conditions, ten tobacco plants showing clear symptoms of bacterial wilt caused by *R. Solanacearum* were selected as the diseased group (BW), and ten asymptomatic, healthy plants from the same field were selected as the control group (CK). Plants were carefully excavated, and loose soil was gently shaken off. The soil tightly adhering to the root surface was defined as rhizosphere soil. For each group, ten rhizosphere subsamples were collected and then pooled and homogenized. The composite sample was subsequently divided into six biological replicates. All samples were placed in sterile sealed bags, transported on ice (~4°C), and stored at –80°C until DNA extraction.

### 2.2 DNA extraction and metagenomic sequencing

Microbial DNA was extracted using the E.Z.N.A.^®^ Fecal DNA Kit (D4015-02, Omega, USA) following the manufacturer’s protocol. Blank controls (unused sterile swabs) were processed alongside the samples to confirm the absence of contaminating DNA. DNA was eluted in 50μl of elution buffer and stored at –80°C until further processing. For library preparation, extracted DNA was enzymatically fragmented using dsDNA Fragmentase (NEB, M0348S) at 37°C for 30minutes. Libraries were constructed using the TruSeq Nano DNA LT Library Preparation Kit (Illumina, USA). Fragmented DNA was end-repaired, A-tailed, and ligated to indexed adapters with T overhangs, which are compatible with A-tailed fragments and contain full-length sequencing primer binding sites for both single- and paired-end reads. Size selection was performed using magnetic beads provided in the kit. Following adapter ligation, libraries were PCR-amplified using the following thermal cycling conditions: 95°C for 3min; 8 cycles of 98°C for 15s, 60°C for 15s, and 72°C for 30s; and a final extension at 72°C for 5min. Sequencing was performed on the Illumina NovaSeq™ 6000 platform using the 2×150 bp paired-end mode, generating approximately 9.0–11.8 Gb of raw data per sample. Raw reads were subjected to quality control using the Read_QC module in MetaWRAP (v0.7). Adapter sequences were removed and low-quality bases with Phred scores < 20 were trimmed using Trim Galore, and reads shorter than 20 bp were discarded. As the samples consisted of environmental soil samples and were not expected to contain contamination from a known host genome, the host-removal step was omitted during quality control. The resulting high-quality reads were used for downstream metagenomic assembly. Sequencing coverage was evaluated by mapping quality-filtered reads back to the assembled contigs using Bowtie2. The results indicated that the majority of assembled sequences were supported by average coverage depths ranging from 6× to 9×, suggesting sufficient sequencing depth for reliable metagenomic assembly and subsequent functional gene prediction. The raw metagenomic sequencing data have been deposited in the NCBI Sequence Read Archive (SRA) under BioProject accession number PRJNA1283476 and are publicly available at https://www.ncbi.nlm.nih.gov/bioproject/PRJNA1283476.

### 2.3 Metagenomic analysis

16S rRNA gene fragments were extracted from quality-filtered metagenomic data using phyloFlash (Gruber-Vodicka et al., 2020) and taxonomically classified using the SILVA database (v138.1) (Quast et al., 2012). Total read counts were calculated for each taxonomic group. Microbial community analyses, including alpha and beta diversity and statistical comparisons, were performed using the microeco (Liu et al., 2020) R package (v4.1.2). Differentially abundant taxa at the genus level were identified using LEfSe (Linear Discriminant Analysis Effect Size) (Segata et al., 2011). Metagenomic assembly was performed using the Assembly module of MetaWRAP (v0.7), retaining only contigs longer than 1,000bp. Open reading frames (ORFs) were predicted using Prokka (Seemann, 2014), and the resulting coding sequences (CDS) were clustered into a non-redundant gene catalog using CD-HIT (v4.6.1) (Li and Godzik, 2006). Gene abundance was quantified using Salmon (Patro et al., 2017), and expression values were normalized as transcripts per million (TPM). Differential gene abundance analysis between healthy and diseased rhizosphere samples was conducted using the limma package (Ritchie et al., 2015) in R (v4.2.2; limma v3.54.2). Differentially abundant genes were identified using thresholds of |log2 fold change| > 2 and FDR–adjusted p value < 0.05, with multiple testing correction performed using the Benjamini–Hochberg method. Functional annotation of the non-redundant gene set was performed using eggNOG-mapper (Huerta-Cepas et al., 2017) with the KEGG database (Kanehisa et al., 2015). KEGG pathway enrichment analysis of differentially abundant genes was performed using TBtools (Chen et al., 2020) to evaluate functional shifts between the two microbial communities. Taxonomic annotation of the gene catalog was performed using the NR database (Pruitt et al., 2005), and genus-level composition within selected functional pathways was determined based on gene abundance profiles.

### 2.4 Co-occurrence network construction

Microbial and gene co-occurrence networks were constructed using the microeco and WGCNA (Langfelder and Horvath, 2008) packages in R. SparCC correlation coefficients (r) were calculated between the abundance of QS functional genes (present in ≥50% of samples and with relative abundance >0.5%) and the abundance of the pathogen genus *Ralstonia*. Only strong (|r| > 0.5) and statistically significant (p < 0.05) correlations were retained to construct the network. Network visualization and modular analysis were conducted in Gephi 0.9.2 (Bastian et al., 2009). Topological properties including the number of nodes and edges, average path length, network diameter, mean node degree, graph density, clustering coefficient, betweenness centrality, and modularity were computed to characterize the structure and complexity of each network. As the network analysis was based on composite biological replicates generated by pooling multiple rhizosphere subsamples, the inferred correlations primarily reflect dominant and reproducible interaction patterns at the condition level, while fine-scale sample-to-sample variability may be underrepresented.

## 3 Result

### 3.1 Microbial community composition in diseased and healthy plants

**Figure 1:**
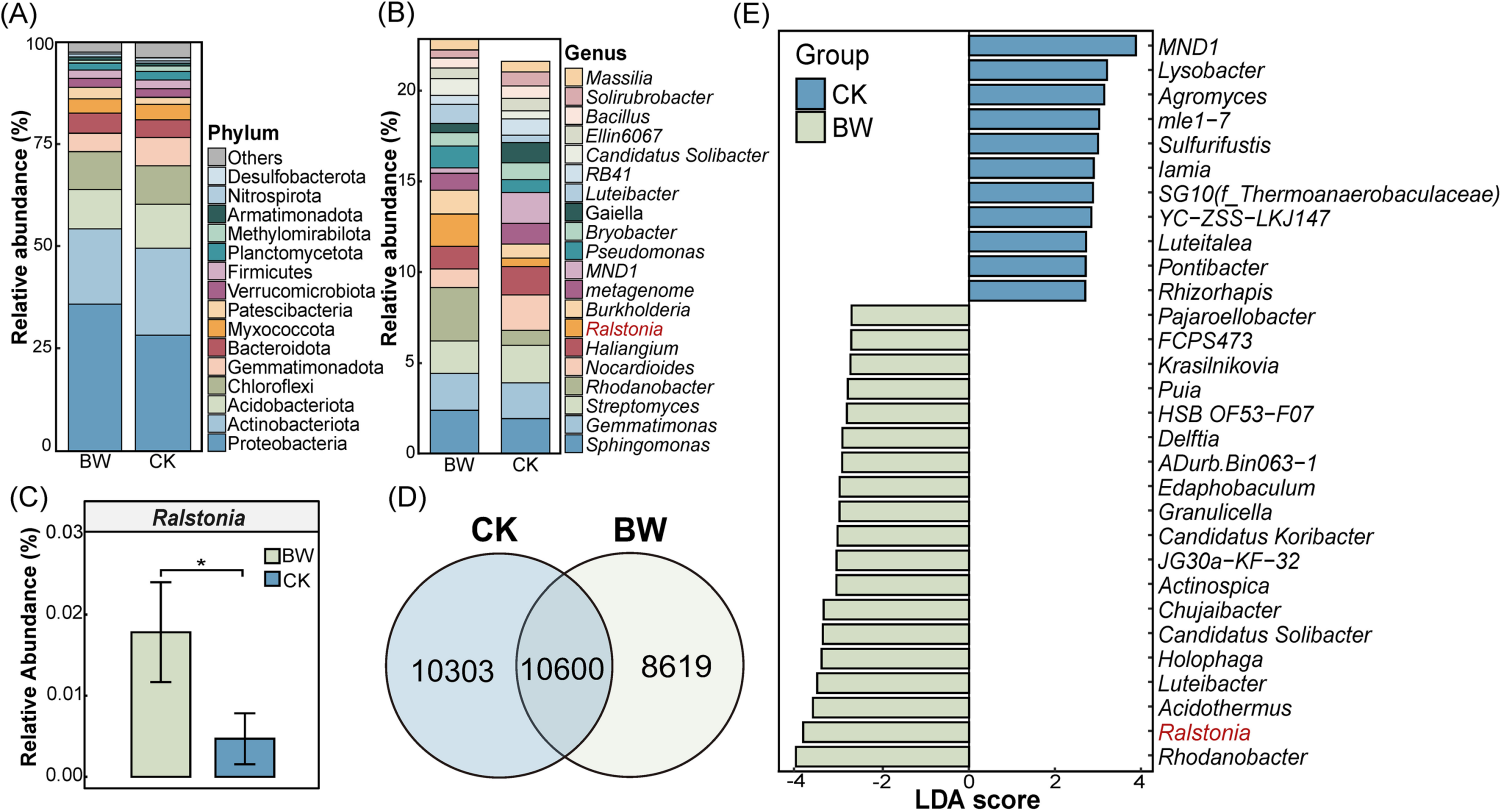
Composition of rhizosphere microbial communities in healthy (CK) and diseased (BW) plants. **(A)** Top 10 most abundant bacterial phyla based on relative abundance. **(B)** Top 20 most abundant bacterial genera based on relative abundance. **(C)** Relative abundance of the pathogen genus *Ralstonia* across treatments. Asterisks indicate significant differences between treatments based on t-test (*p* < 0.05). **(D)** Venn diagram showing shared and unique OTUs between treatment groups. **(E)** Top 30 genera enriched in each treatment group based on LDA scores from LEfSe analysis (*p* < 0.05).

To investigate how bacterial wilt affects the composition of the rhizosphere microbial community, we compared microbial profiles between plants with bacterial wilt (BW) and healthy plants (CK). At the phylum level, the dominant taxa in both BW and CK groups (**Figure 1A**) included Proteobacteria, Actinobacteriota, Acidobacteriota, Chloroflexi, Gemmatimonadota, Bacteroidota, Myxococcota, Patescibacteria, Verrucomicrobiota, and Firmicutes, collectively accounting for more than 90% of the total relative abundance.

At the genus level, the top 20 most abundant genera are shown in **Figure 1B**. Major genera included *Massilia*, *Solirubrobacter*, *Bacillus*, *Ellin6067*, *Luteibacter*, *Gaiella*, *Bryobacter*, *Pseudomonas*, *MND1*, *Burkholderia*, *Ralstonia*, *Haliangium*, *Nocardioides*, *Rhodanobacter*, *Streptomyces*, *Gemmatimonas*, and *Sphingomonas*. Notably, the pathogen genus *Ralstonia* was significantly more abundant in the BW group compared to the CK group (*p* < 0.05) (**Figure 1C**). A Venn diagram (**Figure 1D**) was used to illustrate the overlap and divergence of operational taxonomic units (OTUs) between the two groups. The overlapping region represents OTUs shared by both BW and CK samples (10,600 OTUs), while the unique regions indicate 8,619 BW-specific OTUs and 10,303 CK-specific OTUs.

To further examine taxonomic differences, linear discriminant analysis effect size (LEfSe) was performed at the genus level to identify significantly differentially abundant taxa between groups. **Figure 1E** displays the top 30 genera with significant differences (*p* < 0.05) ranked by LDA score. Genera significantly enriched in the BW group included *Rhodanobacter*, *Ralstonia*, *Acidothermus*, *Luteibacter*, *Holophaga*, *Chujaibacter*, *Actinospica*, *Granulicella*, *Edaphobaculum*, *Delftia*, *Puia*, *Krasilnikovia*, and *Pajaroellobacter*. In contrast, *Rhizorhapis*, *Pontibacter*, *Luteitalea*, *Iamia*, *Sulfurifustis*, *Agromyces*, *Lysobacter*, and *MND1* were significantly enriched in the CK group.

### 3.2 Functional genes profiles of diseased and healthy rhizospheres

**Figure 2:**
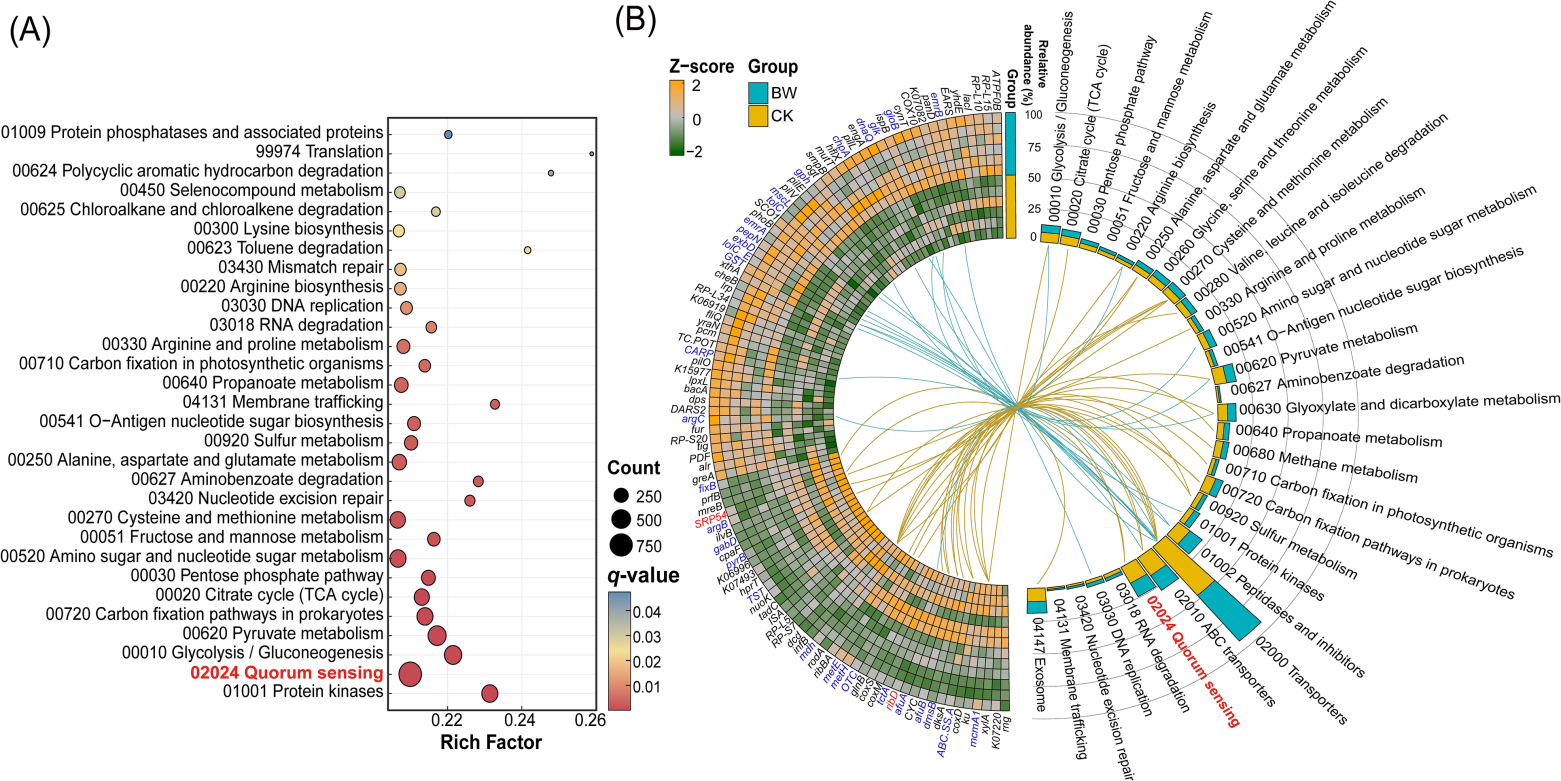
Differential functional gene and pathway profiles in the rhizosphere metagenomes of healthy and diseased plants. *p*-values were adjusted for multiple comparisons using the Benjamini-Hochberg procedure (*q*-value). **(A)** Top 30 KEGG pathways enriched in differential genes, ranked by *p*-value. **(B)** Left: heatmap showing the abundance distribution of the top 100 differentially abundant functional genes across samples (*p* < 0.05). Right: bar plot showing the relative abundance of genes associated with the top 30 significantly enriched KEGG pathways. Connecting lines indicate associations between KEGG Orthology (KO) terms and pathways, with line colors representing higher abundance in either BW or CK.

To explore differences in functional gene profiles between the rhizosphere microbiomes of diseased and healthy plants, we identified differentially abundant genes with logFC > 2 and *P* < 0.05 between the BW (diseased) and CK (healthy) groups. A total of 50,960 such genes were detected. Based on this gene set, KEGG functional enrichment analysis was performed to evaluate their functional roles and enrichment levels. In total, 46 KEGG level 3 pathways were significantly enriched (*P* < 0.05, *q* < 0.2), and the top 30 pathways (ranked by *P*-value) were visualized in a bubble plot (**Figure 2A**). Each bubble represents the number of differentially abundant genes (Gene Number) and the enrichment factor (Rich Factor), indicating the degree of overrepresentation relative to the background gene set. The five most significantly enriched pathways we*re* Protein kinases, Quorum sensing, Glycolysis/Gluconeogenesis, Pyruvate metabolism, and Carbon fixation pathways in prokaryotes. Notably, the Quorum sensing pathway contained the highest number of differentially abundant genes (954) and exhibited a high enrichment level (Rich Factor = 0.21), indicating that QS systems may undergo remodeling during disease development.

To further characterize the distribution of key functional genes, we analyzed the top 100 most abundant differentially expressed KEGG Orthologs (KOs), selected for their representative functional contributions (**Figure 2B**). Among them, 57 KOs were significantly enriched in the BW group, while 43 were more abundant in CK. The most enriched KO in BW was K00799 (*GST*, glutathione S-transferase), with a relative abundance of 0.069%, primarily associated with the Transporters pathway. In contrast, the most abundant KO in CK was K00340 (nuoK, NADH-quinone oxidoreductase subunit K), with a relative abundance of 0.064%, enriched in the Oxidative phosphorylation pathway, which is involved in energy metabolism. Among these 100 high-abundance KOs, two QS-related genes—K03106 (SRP54, signal recognition particle subunit SRP54) and K11752 (*ribD*, diaminohydroxy phosphoribosyl aminopyrimidine deaminase/5-amino-6-(5-phosphoribosylamino) uracil reductase)—were both enriched in the CK group, suggesting a potentially more active expression of QS-associated functions in the rhizosphere of healthy plants. Among these top 100 high-abundance KOs, genes related to the QS system included K03106 (*SRP54*, signal recognition particle subunit SRP54) and K11752 (*ribD*, a bifunctional deaminase/reductase involved in riboflavin biosynthesis), both of which were enriched in the CK group. This indicates that the rhizosphere microbiome of healthy plants may exhibit more active quorum sensing-associated gene potential.

### 3.3 Association between the quorum sensing system and pathogen

**Figure 3:**
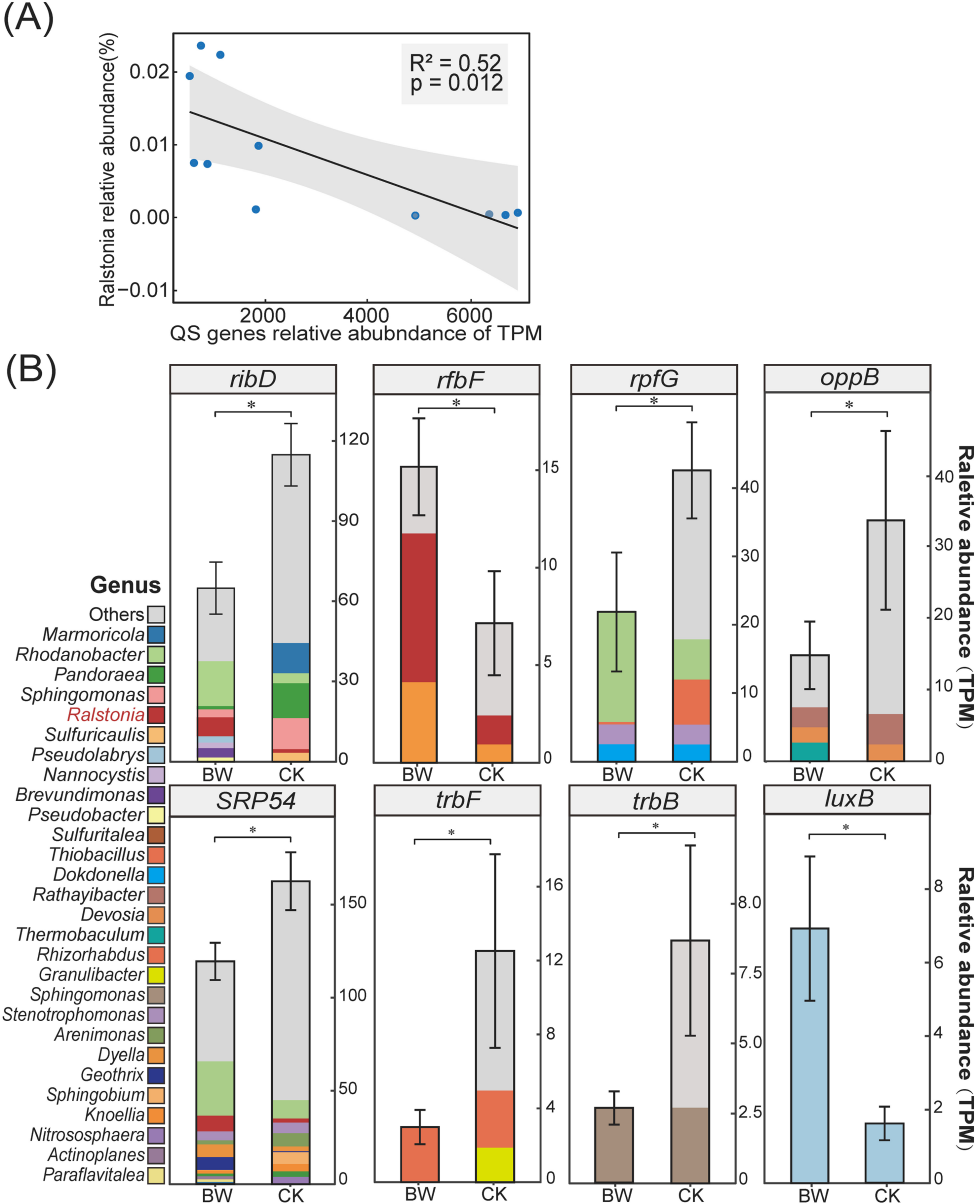
Relationship between QS functional genes and *Ralstonia* abundance, and taxonomic composition of differential QS genes. **(A)** Linear regression analysis between total QS functional gene abundance (TPM) and relative abundance of the genus *Ralstonia* (R² = 0.52, *p* = 0.012). **(B)** Relative abundance of eight significantly different QS-related KO terms between BW and CK. Bar colors represent genus-level taxonomic composition. Asterisks denote significant differences between treatments (*p* < 0.05).

To further investigate the potential regulatory role of the QS system in bacterial wilt, we examined the correlation between the abundance of QS-related genes and the relative abundance of the bacterial wilt pathogen genus *Ralstonia* (**Figure 3A**). A moderate negative correlation was observed (R² = 0.52, *P* < 0.05), indicating that samples with higher *Ralstonia* abundance tend to have a lower overall abundance of QS-related functional genes.

To identify key functional genes contributing to the differences in QS between treatment groups, we screened all KOs annotated to the QS pathway in the KEGG database and compared their abundances between BW and CK groups using a t-test. Eight QS-related genes showed significant differences (**Figure 3B**): K11752 (*ribD*, diaminohydroxyphosphoribosylaminopyrimidine deaminase/5-amino-6-(5-phosphoribosylamino)uracil reductase), K03106 (*SRP54*, signal recognition particle subunit SRP54), K13815 (*rpfG*, two-component system response regulator RpfG), K15581 (*oppB*, oligopeptide transport system permease protein), K20531 (*trbF*, type IV secretion system protein TrbF), K20527 (*trbB*, type IV secretion system protein TrbB), K12990 (rfbF, rhamnosyltransferase), and K15854 (*luxB*, alkanal monooxygenase beta chain). Among them, *ribD, SRP54, rpfG, oppB, trbF*, and *trbB* were more abundant in CK, while *rfbF* and *luxB* were enriched in BW.

To explore the potential microbial sources of these differentially abundant QS-related genes, we annotated the genus-level taxonomic composition of the eight KOs. The pathogen genus *Ralstonia* was among the contributing taxa for *ribD*, *rfbF*, and *SRP54*, and its contribution to these genes was consistently higher in BW than in CK. Specifically, among the dominant taxa associated with *ribD*, *Ralstonia* accounted for 13.73% in the BW group and 1.61% in the CK group. For *SRP54*, *Ralstonia* accounted for 8.13% in BW and 1.99% in CK. In the case of *rfbF*, *Ralstonia* represented 50.49% of the associated taxa in BW and 33.48% in CK. These findings suggest that although QS-related genes were generally more abundant in CK, the involvement of *Ralstonia* in QS gene expression may be more pronounced in diseased plants. This implies that *Ralstonia* could engage in QS-mediated interactions within the rhizosphere microbiome, potentially contributing to disease development. The remaining five significantly different KOs were mainly derived from non-pathogenic bacterial genera, with detailed taxonomic distributions shown in **Figure 3B**.

### 3.4 Quorum sensing genes in the pathogen *R. Solanacearum*

**Figure 4:**
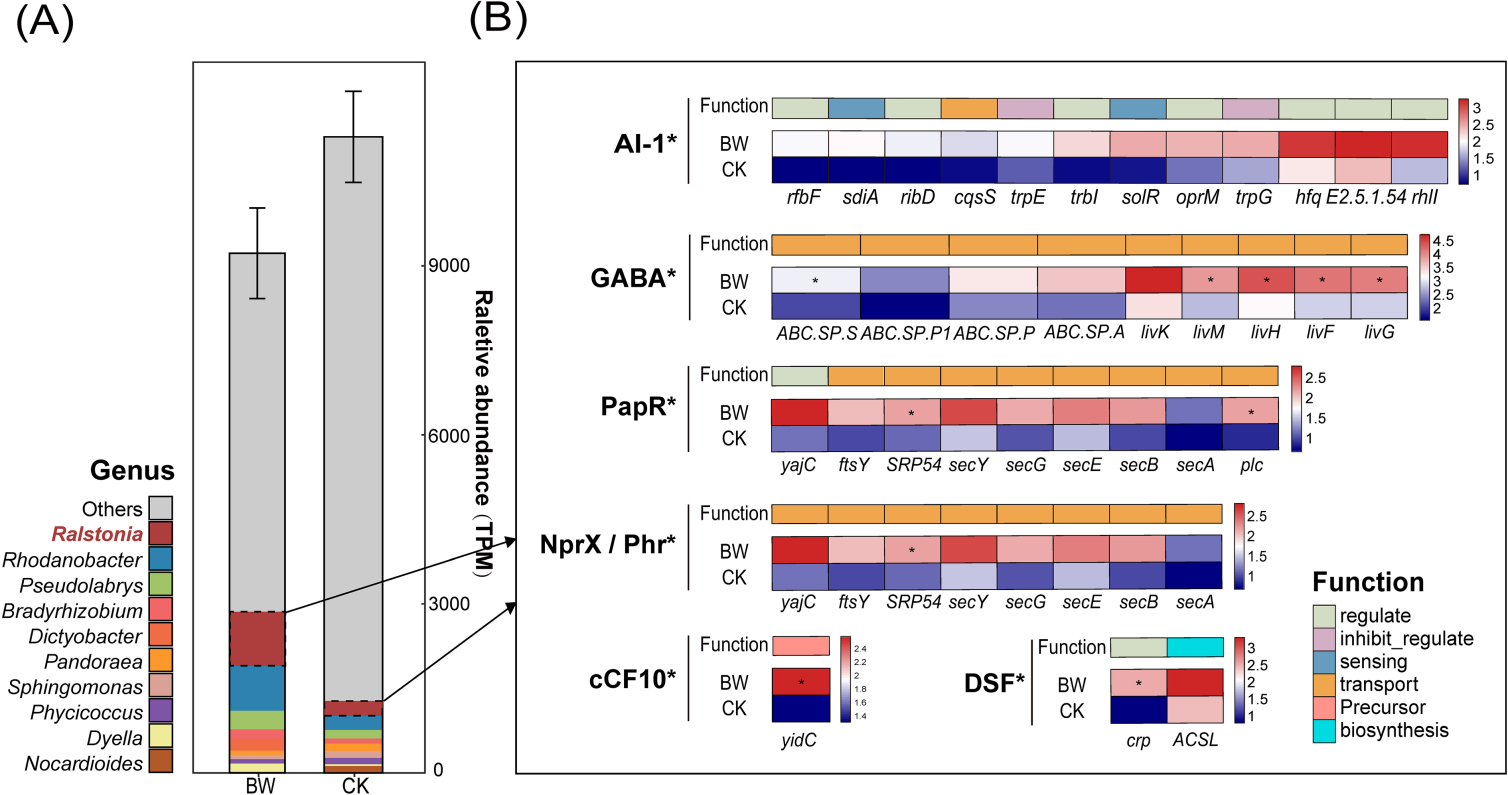
Taxonomic annotation and differential abundance of QS pathway genes contributed by pathogenic genera. **(A)** Left: relative abundance of all QS pathway genes across treatments, annotated with contributing genera. Bar colors represent different genera. Right: heatmap showing the abundance of *Ralstonia*-attributed QS functional genes across different signaling molecule-mediated pathways, limited to those showing significant intergroup differences. Gene function categories are based on KEGG annotations, and indicated by color. Asterisks denote statistically significant differences in gene abundance between BW and CK (*p* < 0.05).

To characterize the composition of QS systems within the rhizosphere microbial community, we identified QS-related functional genes based on KEGG annotations, which represent well-characterized and widely conserved components of quorum sensing systems across diverse bacterial taxa, and subsequently analyzed their genus-level taxonomic sources. In total, 293 bacterial genera were identified as contributors to quorum sensing pathways in rhizosphere soil microbiota. The top 10 genera with the highest gene abundance were visualized (**Figure 4**, left). Notably, the pathogen genus *Ralstonia* was the dominant contributor to QS pathway genes in both BW and CK groups, accounting for 2.33% in CK and increasing to 10.41% in BW.

We further focused on QS pathway genes specifically attributed to the pathogen genus *Ralstonia* and analyzed their differential abundance across QS subsystems classified by distinct signaling molecules (**Figure 4**, right). The results revealed a markedly increased abundance of *Ralstonia*-derived functional genes in BW across multiple QS subsystems, including those mediated by AI-1, GABA, PapR, NprX, Phr, cCF10, and DSF. Specifically, in the AI-1–mediated quorum sensing pathway, the overall abundance of functional genes was significantly higher in BW than in CK, although no individual gene showed statistically significant differences. In the GABA-mediated pathway, five genes—K02055 (*ABC.SP.S*), K01998 (*livM*), K01997 (*livH*), K01996 (*livF*), and K01995 (*livG*)—were significantly more abundant in the BW group.

In the PapR-mediated QS pathway, K03106 (*SRP54*, signal recognition particle subunit SRP54) and K01114 (*plc*, phospholipase C) were both significantly enriched in BW. The signaling molecules NprX and Phr shared identical annotated genes, among which only *SRP54* exhibited significant abundance differences. Interestingly, while *SRP54* showed higher abundance in CK when considering the entire microbial community, it displayed the opposite trend—being more enriched in BW—when restricted to genes attributed specifically to pathogens.

In the cCF10-mediated pathway, only one pathogenic gene, K03217 (*YidC*, family membrane protein insertase), was detected, and it exhibited a significant difference between BW and CK. In the DSF-mediated pathway, K10914 (*crp*, family transcriptional regulator, cyclic AMP receptor protein) also showed significant abundance variation.

### 3.5 Co-occurrence network analysis of QS genes and pathogen abundance

**Figure 5:**
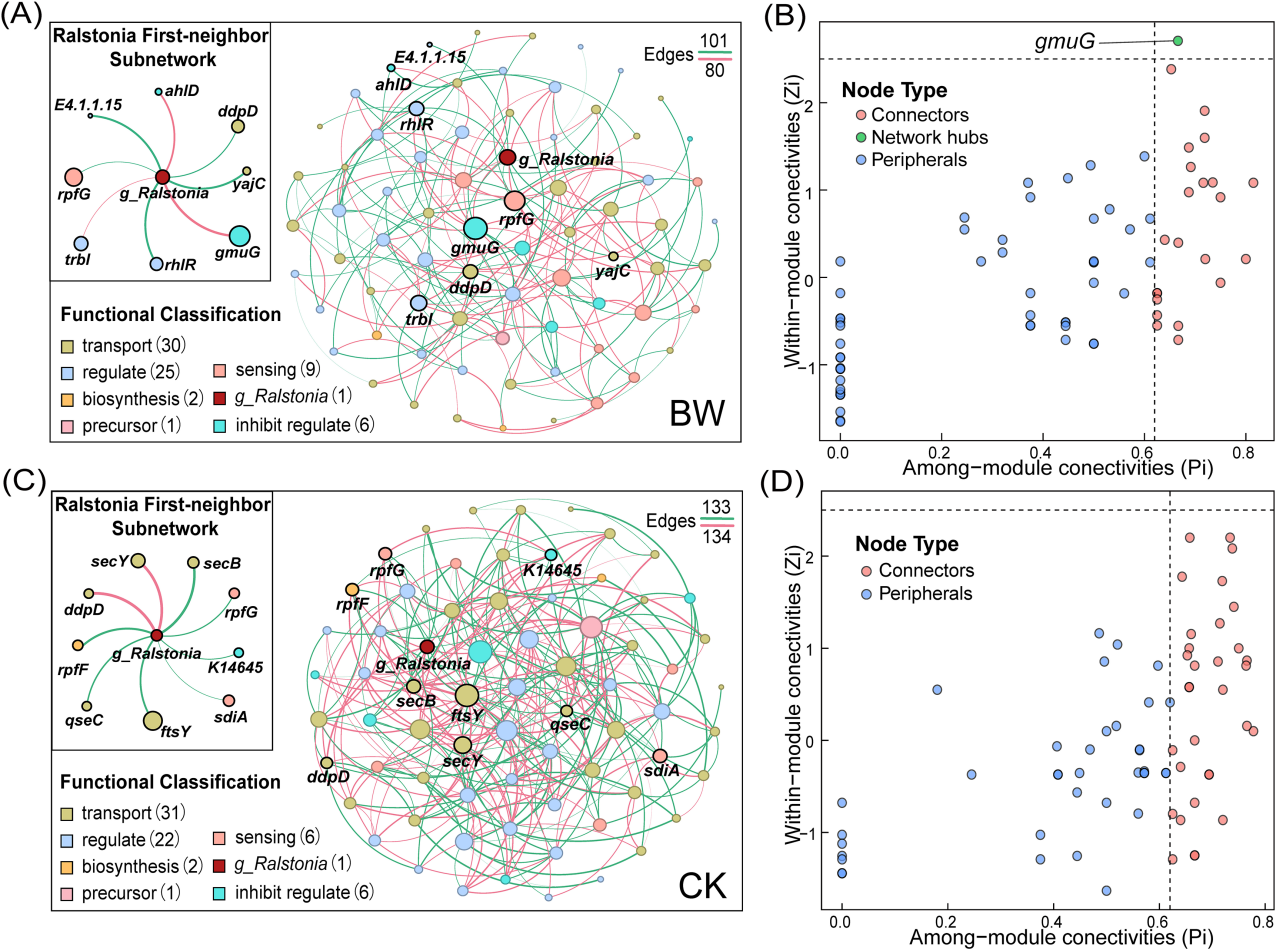
Co-occurrence networks and *Z_i_*–*P_i_* topological roles of QS functional genes and *Ralstonia* across treatments. **(A)** Co-occurrence network of QS functional genes and *Ralstonia* in the BW group inferred using the SparCC algorithm. The full network is shown together with a subnetwork comprising nodes directly connected to *Ralstonia*. Nodes represent QS functional genes or *Ralstonia*, and edges indicate significant correlations (|r| > 0.5, *p* < 0.05). Node colors denote functional categories, and edge colors indicate correlation direction (red, positive; green, negative). **(B)***Z_i_*–*P_i_* plot showing the topological roles of nodes in the BW network corresponding to **(A)**. Nodes are classified into four categories based on within-module connectivity (*Z_i_*) and among-module connectivity (*P_i_*): network hubs (*Z_i_* > 2.5, *P_i_* > 0.62), module hubs (*Z_i_* > 2.5, *P_i_* ≤ 0.62), connectors (*Z_i_* ≤ 2.5, *P_i_* > 0.62), and peripherals (*Z_i_* ≤ 2.5, *P_i_* ≤ 0.62). **(C)** Co-occurrence network of QS functional genes and *Ralstonia* in the CK group, constructed and visualized using the same criteria as in **(A)**. **(D)***Z_i_*–*P_i_* plot showing the topological roles of nodes in the CK network corresponding to **(C)**, classified using the same *Z_i_* and *P_i_* thresholds as in **(B)**.

To investigate the potential relationship between the pathogen genus *Ralstonia* and QS system functions, we constructed co-occurrence networks between QS-related functional genes and the abundance of *Ralstonia* using the SparCC algorithm (**Figure 5**). The topological properties of the networks under different treatments are summarized in **Supplementary Table S1**, while the node-level topological roles based on *Z_i_ - P_i_* classification are provided in **Supplementary Tables S3** and **S4**. The CK network comprised 69 nodes and 267 edges, while the BW network included 74 nodes and 181 edges, indicating tighter connections among functional genes in CK. The network density in CK (0.114) was substantially higher than that in BW (0.067), suggesting a more complex functional network structure in the healthy group. The key hub node, K01218 (*gmuG*, mannan endo-1,4-beta-mannosidase), was identified in the BW network, while no hub-like nodes were detected in CK. However, the number of connector nodes in CK (32) greatly exceeded that in BW (22).

From a functional category perspective, BW and CK showed slight differences in the number of genes involved in transport (30 vs. 31) and regulation (25 vs. 22), while other categories were largely comparable. Notably, the two networks exhibited clear differences in nodes directly connected to *Ralstonia*. In BW, three genes annotated with regulatory functions—K20533 (*trbI*, type IV secretion system protein), K18099 (*rhlR*, LuxR family transcriptional regulator), and K01580 *(GAD*, glutamate decarboxylase)—were directly linked to *Ralstonia*. Among them, *trbI* showed a positive correlation, while the other two were negatively correlated. In contrast, no regulate-type genes were directly connected to *Ralstonia* in the CK network. Regarding inhibit-regulate functions, two genes—*gmuG* (the identified hub) and K13075 (*ahlD*, N-acyl homoserine lactone hydrolase)—were positively correlated with *Ralstonia* in BW. In CK, only one such gene, K14645 (*K14645*, serine protease), was detected, which showed a negative correlation with *Ralstonia*. Additionally, a biosynthesis-related gene K13816 (*rpfF*, DSF synthase) was directly linked to *Ralstonia* in CK with a negative correlation, whereas no such association was found in BW. The sensing-related gene K13815 (*rpfG*, two-component response regulator) was negatively correlated with *Ralstonia* in both networks. Another sensing gene, K07782 (*sdiA*, LuxR family regulator), was also negatively correlated with *Ralstonia* in CK. Furthermore, two transport-associated genes—K02031 (*ddpD*, peptide/nickel transport ATP-binding protein) and K03210 (*yajC*, preprotein translocase subunit)—were directly connected to *Ralstonia* in BW and both exhibited negative correlations.

## 4 Discussion

In this study we found that *R. Solanacearum* interferes with community quorum sensing by enhancing its relative dominance within the QS system. Analysis of the rhizosphere microbial community composition revealed a significant increase in the relative abundance of the pathogen genus *Ralstonia* in the diseased group (**Figure 1C**), while several potentially beneficial genera, including *Streptomyces, Rhizorhapis*, and *MND1*, were significantly enriched in the healthy group (**Figure 1E**). However, diversity analyses showed no significant differences in α-diversity between healthy and diseased plants (**Supplementary Table S2**), indicating that the overall microbial community structure remained relatively stable despite shifts in dominant taxa. Against this backdrop of structural stability, we observed marked changes in the QS system of the rhizosphere microbiome. Specifically, KEGG functional enrichment analysis revealed that the QS pathway contained the largest number of differentially expressed genes and showed a relatively high enrichment level (**Figure 2A**), exceeding most metabolism-related pathways. This suggests that the QS system is highly responsive to disease pressure and may play a crucial regulatory role in disease onset. Such functional shifts under structurally stable community frameworks may represent an efficient ecological strategy by which pathogens alter community behavior without displacing the entire microbiome.

Further analysis revealed a significant negative correlation between the overall abundance of QS-related functional genes in the rhizosphere microbiome and the relative abundance of the pathogen genus *Ralstonia* (**Figure 3A**). It suggests that under conditions of extensive *Ralstonia* colonization, the pathogen may suppress or disrupt the QS expression capacity of other community members through specific mechanisms. To further investigate functional alterations in the QS system, we identified eight QS-related genes that were significantly differentially abundant between the rhizosphere of diseased and healthy plants: *ribD, SRP54, rpfG, oppB, trbF, trbB, rfbF*, and *luxB*.

These genes participate in multiple key steps of the QS signaling cascade. *SRP54* and *rpfG* are involved in signal recognition and response regulation, serving as critical nodes for QS sensin (Dow, 2008); *ribD* contributes to the biosynthesis of QS signal precursors; *oppB* (Doeven et al., 2004), *trbF*, and *trbB* (Alt-Mörbe et al., 1996) encode transmembrane proteins associated with peptide transport and secretion systems, which may be essential for the effective transmission of QS signals. The enrichment of these genes in the rhizosphere of healthy plants indicates that, in undisturbed microbial ecosystems, the QS pathway preserves an intact “recognition–perception–response” cascade, supporting coordinated signaling and symbiotic equilibrium within the microbial community. Such an intact QS signaling cascade is likely critical for maintaining cooperative behaviors, resource sharing, and antagonistic balance among rhizosphere microbes, thereby contributing to ecological stability and resistance against pathogen invasion. In contrast, *rfbF*—enriched in the rhizosphere of diseased plants—is involved in extracellular polysaccharide (EPS) biosynthesis (Rahim et al., 2001), while *luxB* contributes to the synthesis of AHL-type QS signals (Swartzman et al., 1992). The increased abundance of these genes under pathogen-dominated conditions suggests heightened activity of QS functions associated with biofilm formation. This shift in QS dynamics aligns with a major virulence strategy of *R. Solanacearum*—biofilm development (An and Zhang, 2024). Therefore, the functional skew of community QS genes may facilitate the creation of a microenvironment favorable for pathogen persistence and proliferation. This functional skew suggests a transition from a community-level communicative equilibrium to a pathogen-favored signaling environment, in which QS-mediated cooperation is repurposed to support biofilm formation and localized dominance. It should be noted that SRP54 and ribD, identified here as QS-related genes, are multifunctional housekeeping genes, and their association with quorum sensing is based on KEGG pathway annotations. Variations in their abundance may therefore be influenced by additional regulatory or metabolic factors. Nevertheless, the overall enrichment of the QS pathway, together with coordinated abundance shifts in other QS-associated functional genes, supports the broader conclusion that QS-related functions are restructured during disease development.

Taxonomic annotation of these differential QS genes further revealed dynamic changes in their microbial sources during disease progression. In healthy rhizospheres, the QS genes were primarily contributed by well-known probiotic genera such as *Lysobacter* and *Pseudomonas*, which exhibit strong metabolic regulatory capacities and potential for mutualistic plant interactions (Zboralski et al., 2020). In contrast, in the rhizosphere of diseased plants, the pathogen genus *Ralstonia* progressively became the dominant contributor of QS-related genes. This shift in signaling origin may lead to the collapse of the originally multi-nodal, cooperative QS network into a pathogen-dominated, unipolar signaling system, ultimately destabilizing microbial communication homeostasis. Ecologically, this shift may reduce functional redundancy and weaken the buffering capacity of the rhizosphere microbiome, making the system more vulnerable to environmental fluctuations and pathogen-induced perturbations.

Previous studies have shown that members of the *R. Solanacearum* species complex (RSSC) possess multiple QS circuits. In addition to the classical phcBSR system (Kai, 2023), strain EP1 encodes a LuxI/LuxR homolog pair (RasI/RasR), in which RasI synthesizes 3-hydroxy-dodecanoyl homoserine lactone (3-OH-C12-HSL) (Clough et al., 1997), regulating over 150 genes related to metabolism and virulence. The RasI/RasR system is crucial for extracellular enzyme production, motility, biofilm formation, and virulence expression (Yan et al., 2022). In the present study, multiple observations collectively point to an enhanced involvement of the pathogen genus *Ralstonia* in QS-associated functions under diseased conditions. These include the strong representation of the QS pathway among differentially abundant functions, the enrichment of specific QS-related genes linked to signal production and biofilm formation, and the progressive dominance of the pathogen genus *Ralstonia* as a contributor to QS-related genes at the community level. Together, these observations are consistent with a scenario in which *R. Solanacearum* increases its relative influence within the community QS landscape, potentially shifting signal production and response dynamics in its favor. Such changes may coincide with a reduced representation of QS sensing and signaling components contributed by other microbial taxa, thereby constraining community-level signal perception and coordinated responses. Further investigations focused on QS functional genes dominated by *R. Solanacearum* to reveal potential mechanisms by which they may interfere with the community QS systems (**Figure 4A**). In our study, the overall abundance of AI-1 pathway-related QS genes contributed by the pathogen was significantly increased in the rhizosphere of diseased plants. Although no single gene showed significant differences, this suggests that *R. Solanacearum* may regulate the AHL pathway through synergistic actions of multiple genes, rather than relying on a specific target. *R. Solanacearum* harbors the SolI/SolR AHL synthesis-sensing system, in which SolR functions as an AHL-responsive transcriptional regulator (Zuluaga Cruz et al., 2013). Previous studies have shown that this pathogen can synthesize AHL signal molecules to regulate virulence expression (Kai, 2023), and can also degrade exogenous AHL signals via AHL lactonase (Kalia, 2013), thereby quenching QS signaling in competing bacteria. However, in our study, the gene abundance of AHL lactonase was not significantly increased.

In the γ-aminobutyric acid (GABA) signaling pathway, several genes encoding polyamine and branched-chain amino acid transport proteins (e.g., ABC.SP.S, *livK*, *livM*, *livH*, *livF*, *livG*) were significantly upregulated in diseased samples. These genes belong to the spermidine/putrescine ABC transport system (Adams et al., 1990), which is closely related to GABA uptake. GABA, secreted by plants, is taken up by bacteria and converted into succinic semialdehyde (SSA), which inhibits the activity of the QS-related transcription factor BlcR, thereby relieving its repression on *blcC* and promoting AHL degradation via BlcC (Chevrot et al., 2006). Prior studies suggest that *R. Solanacearum* can manipulate plant metabolism to increase the production of GABA and polyamines, which not only serve as nutrient sources supporting colonization in xylem (Wang et al., 2019; Zhao et al., 2025), but also indirectly interfere with AHL-based QS in other microbes. GabT (GABA transaminase) has also been confirmed as a key factor in virulence regulation of *R. Solanacearum* (Xian et al., 2021). Thus, *R. Solanacearum* may simultaneously exploit the GABA pathway to promote its own proliferation and suppress interspecies AHL signaling, gaining ecological advantage.

The PapR/NprX/Phr peptide-based QS pathway, commonly found in Gram-positive bacteria (Grenha et al., 2013), showed significant upregulation of only *plc* (phospholipase C) (Ostroff et al., 1990) and *SRP54* in the diseased group. *SRP54* is involved in the translocation of QS peptides into the extracellular environment, while *plc*, through phosphorylation/dephosphorylation mechanisms, regulates downstream QS responses at structural and functional levels (Hawver et al., 2016). Their upregulation suggests that *R. Solanacearum* may enhance peptide signal secretion and prepare to amplify downstream signal transduction.

In the cCF10 peptide signaling pathway, which regulates plasmid conjugation, the membrane protein insertion factor *YidC* was also significantly more abundant in the rhizosphere of diseased plants. *YidC* assists the Sec translocon in inserting peptides into the membrane, thereby influencing the reception and release of QS signal (Fröderberg et al., 2004). This suggests that *R. Solanacearum* may enhance the reception and secretion of such signaling molecules, thereby facilitating plasmid conjugation within the microbial community. Previous studies have shown that *R. Solanacearum* can exploit plasmid-borne genes to evade plant immune responses or transfer virulence factors, thereby enhancing its survival and pathogenic potential (Guidot et al., 2009). Moreover, additional studies have demonstrated that specific *R. Solanacearum* strains have gained novel type III effector genes via horizontal gene transfer, which suppress salicylic acid–mediated plant defenses and contribute to immune evasion and pathogenicity (Landry et al., 2020; Qi et al., 2022; Tan et al., 2019).

Diffusible signal factor (DSF) molecules, a class of unsaturated fatty acid signals produced by phytopathogens (Ryan and Dow, 2011), are also synthesized by *R. Solanacearum* (Baltenneck et al., 2021). Our study identified a significant upregulation of *crp*, a downstream CRP/FNR family transcription factor in the DSF pathway, in diseased samples. The CRP/cAMP system is a classic bacterial regulatory module (Gosset et al., 2004), and its upregulation suggests that *R. Solanacearum* may amplify its response to DSF signals through enriched transcriptional regulators (e.g., Clp), thus enhancing EPS biosynthesis (Deng et al., 2011) and other virulence-regulating mechanism (Tao et al., 2010).

Taken together, our results suggest *R. Solanacearum* may mediate community QS interference through multiple layers of regulation: on one hand, by expressing signal-degrading enzymes (e.g., AHL lactonase) or manipulating plant metabolism (e.g., inducing GABA production) to dampen QS signaling in other microbes and indirectly strengthen its own; on the other hand, by enhancing its own ability to synthesize, secrete, and perceive QS signals, thus promoting EPS production, virulence gene transfer via plasmid conjugation, and ultimately reinforcing its pathogenic capacity while reshaping the rhizosphere microbiome toward a disease-favorable state.

Based on the co-occurrence network analysis between QS functional genes and the pathogen genus *Ralstonia*, we observed pronounced differences in network topology between healthy and diseased rhizosphere samples. Compared with the healthy rhizosphere, which was characterized by higher network density, the diseased state displays a simpler network structure, characterized by reduced connectivity, diminished modularity, and fewer key bridging nodes. From a network ecology perspective, reduced connectivity and modularity may reflect a loss of cooperative interactions and functional compartmentalization, both of which are important for maintaining microbial community resilience. Such changes may reflect a disturbance of QS-associated interactions within the rhizosphere microbial community during the development of bacterial wilt. We hypothesize that this network reorganization may be driven by the increasing dominance of the pathogen *R. Solanacearum* within the QS system. By occupying a central position in the community’s QS network, *R. Solanacearum* appears to rewire the pre-existing signaling system, which originally dominated by probiotic genera, shifting signaling authority and breaking pre-established communication pathways. Notably, the gene *gmuG*, which functions downstream of quorum sensing regulation, emerged as a key hub in the diseased network. *gmuG* is not a canonical signal synthesis or perception gene, it is involved in extracellular polysaccharide biosynthesis and is regulated through the DSF–c-di-GMP signaling cascade (Deng et al., 2011; Dow et al., 2003). Its central position within the QS-related co-occurrence network therefore likely reflects an increased coupling between QS signal transduction and downstream biofilm-associated functional under pathogen-dominated conditions. It should be noted that, due to the limited number of biological replicates, the co-occurrence network analysis was performed for exploratory purposes, and the inferred associations require validation in larger datasets.

Among QS-related functional genes directly associated with the pathogen genus *Ralstonia*, *trbI* (encoding a type IV secretion system protein) in the diseased group may participate in QS-mediated virulence factor secretion and plasmid transfer (Torres Tejerizo et al., 2014), whereas *ahlD* (an AHL lactonase) functions in quorum quenching (Dong et al., 2002). Both genes exhibited significant positive correlations with *Ralstonia* within the network, suggesting that *R. Solanacearum* may disrupt community-level QS communication by upregulating quorum-quenching genes, while concurrently enhancing virulence factor secretion through the QS network. In contrast, the regulatory gene *rhlR* and the signal-responsive gene *rpfG* displayed negative correlations with *Ralstonia* in the diseased group, implying that *R. Solanacearum* may suppress QS signal perception in other microbes, thereby weakening their ability to respond to environmental cues. The central gene *gmuG* also showed a positive correlation with *Ralstonia*, potentially indicating that pathogen leverages the QS system to promote biofilm formation as a major QS-mediated function in the rhizosphere community—consistent with its established pathogenic strategies (An and Zhang, 2024).

Co-occurrence network analysis further supports the view that *R. Solanacearum* interferes with the QS signal perception of other microorganisms, disrupting ecological relationships such as resource sharing and antagonistic symbiosis, which are typically coordinated by QS. This interference undermines microbial community homeostasis and creates a niche with reduced competition, facilitating pathogen colonization. Simultaneously, the pathogen-dominated QS system exacerbates community-level functional disorder, enhances overall pathogenic potential, and ultimately shifts microbial community functionality toward a disease-favoring trajectory.

It should be noted that the pooling of multiple subsamples into composite replicates likely reduced stochastic variability in the dataset. Consequently, the inferred co-occurrence associations primarily reflect dominant and reproducible interaction patterns distinguishing healthy and diseased rhizosphere communities. In addition, the identification of QS-related genes and pathways in this study is based on metagenomic annotation and computational inference. Although these approaches provide insights into potential functional patterns at the community level, they do not directly demonstrate gene expression, signal production, or regulatory activity. Therefore, the QS-related associations reported here should be interpreted as putative. Experimental validation will be required in future studies to confirm the specific roles and mechanisms of these QS systems.

Despite these limitations, our study offers conceptual insights that may inform the management of soil-borne diseases in agricultural systems. Rather than targeting individual pathogens directly, QS-based management strategies could aim to disrupt pathogen-dominated signaling networks or restore community-level communication balance in the rhizosphere. For example, enhancing quorum-quenching activities, promoting microbial taxa capable of stabilizing QS interactions, or applying soil amendments that interfere with pathogen-associated QS signals may represent promising ecological approaches for disease suppression. By mitigating QS-driven dominance and virulence expression of pathogens such as *R. Solanacearum*, it may be possible to shift the rhizosphere toward a more stable and disease-suppressive state under field conditions. Nevertheless, translating QS-based ecological management concepts into field applications will require careful consideration of practical constraints, including spatial heterogeneity of soil environments, the stability and diffusion of signaling molecules, and the effective delivery and persistence of quorum-quenching agents under complex rhizosphere conditions.

## 5 Conclusion

This study demonstrates that quorum sensing (QS)-related gene composition, functional pathways, and co-occurrence network structure differ markedly between healthy and bacterial wilt–affected rhizosphere soils. Our data directly support that *R. Solanacearum* occupies a central and dominant position within the QS-related network under diseased conditions, accompanied by pronounced shifts in QS gene abundance and network topology. We further observed the enrichment of pathogen-derived genes (e.g., *rfbF*, *luxB*, and *crp*) and the depletion of key signaling components (e.g., *SRP54* and *ribD*) associated with QS integrity in healthy microbial communities. Together, these observations indicate a reorganization of community-level QS-associated patterns during disease development.

Based on these findings, we propose that *R. Solanacearum* may influence the community QS landscape by enhancing its own QS-related functions while coinciding with a reduced representation of QS sensing and signaling components contributed by other microbial taxa. The observed dominance of pathogen-derived QS genes, together with shifts in metabolite-associated transport pathways, is consistent with a disturbance of cooperative signaling at the community level; however, the specific molecular mechanisms underlying such interference remain to be experimentally resolved.

Collectively, these results provide a theoretical framework for the ecological management of soil-borne diseases. Instead of targeting pathogens in isolation, this approach emphasizes modulating QS-mediated community interactions to suppress disease.

## Data Availability

The datasets presented in this study can be found in online repositories. The names of the repository/repositories and accession number(s) can be found in the article/**Supplementary Material**.
