# Supplementary material for: The role of quorum sensing in rhizosphere community regulation during bacterial wilt pathogen invasion
Source: Front Plant Sci. 2026 Feb 16;17:1685007. doi: 10.3389/fpls.2026.1685007 (PMC12950752; doi:10.3389/fpls.2026.1685007)
Supplement: Supplementary file 1 [file Table1.docx]

**Table S1 Topological properties of the QS gene co-occurrence networks in healthy (CK) and wilt-infected (BW) rhizosphere microbiomes.**

|  | **Vertex** | **Edge** | **Average degree** | **Average path length** | **Network diameter** | **Clustering coefficient** | **Density** | **Heterogeneity** | **Centralization** | **Modularity** |
| --- | --- | --- | --- | --- | --- | --- | --- | --- | --- | --- |
| CK | 69 | 267 | 7.73913 | 1.776754 | 4 | 0.150066 | 0.113811 | 0.475098 | 0.136189 | 0.294311 |
| BW | 74 | 181 | 4.891892 | 2.344614 | 5 | 0.096482 | 0.067012 | 0.576767 | 0.124769 | 0.416048 |

**Table S2. Alpha diversity comparison of rhizosphere microbial communities in healthy and diseased plants.**

|  | **Observed** | **Chao1** | **se.chao1** | **ACE** | **Shannon** | **InvSimpson** | **Fisher** | **Pielou** | **Coverage** |
| --- | --- | --- | --- | --- | --- | --- | --- | --- | --- |
| CK | 5969.50±135.598 | 6768.35±176.968 | 55.73±3.019 | 6867.17±185.867 | 8.16±0.042 | 1810.78±170.226 | 2304.07±81.782 | 0.94±0.003 | 0.95±0.003 |
| BW | 5785.83±206.225 | 6606.67±285.289 | 56.56±3.552 | 6754.45±307.353 | 8.02±0.052 | 1196.79±135.876 | 2200.04±119.682 | 0.93±0.004 | 0.95±0.004 |
| P. value | 0.179654 | 0.24026 | 0.393939 | 0.309524 | 0.309524 | 0.132035 | 0.179654 | 0.24026 | 0.937229 |

**Table S3. *Z*_i_ - *P_i_* based topological classification of QS-related genes and the pathogen genus *Ralstonia* in the wilt-infected (BW) rhizosphere network.**

| **Nodes** | ***Z_i_*** | ***P_i_*** | **Type** |
| --- | --- | --- | --- |
| *plc* | -1.343 | 0.000 | Peripherals |
| *ribA* | -1.343 | 0.000 | Peripherals |
| *E4.1.1.15* | -1.047 | 0.000 | Peripherals |
| *E2.5.1.54* | -0.181 | 0.375 | Peripherals |
| *trpE* | -1.316 | 0.000 | Peripherals |
| *ACSL* | -0.758 | 0.500 | Peripherals |
| *livG* | 0.173 | 0.500 | Peripherals |
| *livM* | -1.640 | 0.000 | Peripherals |
| *livK* | -0.181 | 0.000 | Peripherals |
| *ABC.PE.P* | -1.343 | 0.000 | Peripherals |
| *ABC.PE.P1* | -1.650 | 0.000 | Peripherals |
| *ABC.PE.S* | -0.469 | 0.000 | Peripherals |
| *ABC.SP.P* | -0.515 | 0.444 | Peripherals |
| *ABC.SP.P1* | -0.758 | 0.000 | Peripherals |
| *ABC.SP.S* | -0.183 | 0.560 | Peripherals |
| *flhC* | -0.515 | 0.444 | Peripherals |
| *flhD* | -1.039 | 0.000 | Peripherals |
| *secB* | 1.135 | 0.449 | Peripherals |
| *secE* | 0.183 | 0.278 | Peripherals |
| *secY* | -0.550 | 0.375 | Peripherals |
| *SRP54* | -1.047 | 0.000 | Peripherals |
| *ftsY* | 0.286 | 0.320 | Peripherals |
| *yajC* | -0.062 | 0.500 | Peripherals |
| *hfq* | -0.515 | 0.000 | Peripherals |
| *qseC* | 0.173 | 0.500 | Peripherals |
| *qseB* | 0.673 | 0.500 | Peripherals |
| *kdpE* | 0.431 | 0.320 | Peripherals |
| *spo0A* | 0.173 | 0.611 | Peripherals |
| *glrK* | -0.715 | 0.444 | Peripherals |
| *glrR* | -0.554 | 0.444 | Peripherals |
| *lasA* | -0.433 | 0.375 | Peripherals |
| *slo* | 0.550 | 0.245 | Peripherals |
| *ribD* | -0.916 | 0.000 | Peripherals |
| *rfbF* | 0.183 | 0.000 | Peripherals |
| *rhlI* | -0.916 | 0.000 | Peripherals |
| *ahlD* | -0.469 | 0.000 | Peripherals |
| *rpfF* | -0.550 | 0.375 | Peripherals |
| *K14645* | 0.673 | 0.611 | Peripherals |
| *oppA* | -1.283 | 0.000 | Peripherals |
| *oppB* | -1.178 | 0.000 | Peripherals |
| *oppD* | -0.758 | 0.500 | Peripherals |
| *rhlR* | 0.916 | 0.375 | Peripherals |
| *mexH* | 0.686 | 0.245 | Peripherals |
| *expR* | 1.283 | 0.494 | Peripherals |
| *fusK* | -1.539 | 0.000 | Peripherals |
| *fusR* | 0.780 | 0.531 | Peripherals |
| *gadC* | 0.183 | 0.500 | Peripherals |
| *rgg2* | 1.083 | 0.370 | Peripherals |
| *ropB* | 1.386 | 0.600 | Peripherals |
| *nisR* | 0.550 | 0.571 | Peripherals |
| *trbC* | -0.550 | 0.000 | Peripherals |
| ***gmuG*** | **2.708** | **0.724** | **Network hubs** |
| *livF* | 1.083 | 0.716 | Connectors |
| *livH* | -0.181 | 0.625 | Connectors |
| *ddpD* | 0.975 | 0.688 | Connectors |
| *ddpF* | 1.487 | 0.688 | Connectors |
| *ABC.SP.A* | -0.550 | 0.625 | Connectors |
| *yidC* | 1.087 | 0.735 | Connectors |
| *phzF* | -0.181 | 0.625 | Connectors |
| *sdiA* | -0.252 | 0.625 | Connectors |
| *zur* | -0.062 | 0.750 | Connectors |
| *rpfC* | -0.554 | 0.667 | Connectors |
| *oppF* | 0.431 | 0.640 | Connectors |
| *crp* | 0.397 | 0.667 | Connectors |
| *secDF* | -0.715 | 0.667 | Connectors |
| *rpfG* | 2.383 | 0.653 | Connectors |
| *oppC* | 0.210 | 0.720 | Connectors |
| *solR* | -0.433 | 0.625 | Connectors |
| *phzG* | 1.598 | 0.719 | Connectors |
| *toxD* | 0.210 | 0.800 | Connectors |
| *blpA* | 1.908 | 0.719 | Connectors |
| *lanB* | 0.916 | 0.750 | Connectors |
| *trbI* | 1.083 | 0.815 | Connectors |
| *g_Ralstonia* | 1.264 | 0.691 | Connectors |

**Table S4. *Z*_i_ - *P_i_* based topological classification of QS-related genes and the pathogen genus *Ralstonia* in the healthy (CK) rhizosphere network.**

| **Nodes** | ***Z_i_*** | ***P_i_*** | **Type** |
| --- | --- | --- | --- |
| *plc* | -0.065 | 0.406 | Peripherals |
| *ribA* | 1.041 | 0.521 | Peripherals |
| *E4.1.1.15* | -1.633 | 0.500 | Peripherals |
| *trpG* | 0.549 | 0.180 | Peripherals |
| *pel* | -1.025 | 0.375 | Peripherals |
| *livF* | -1.443 | 0.000 | Peripherals |
| *livH* | -1.443 | 0.000 | Peripherals |
| *livK* | -1.255 | 0.000 | Peripherals |
| *ddpD* | -0.355 | 0.612 | Peripherals |
| *ddpF* | 0.811 | 0.597 | Peripherals |
| *ABC.PE.P* | -0.350 | 0.560 | Peripherals |
| *ABC.PE.P1* | -1.025 | 0.000 | Peripherals |
| *ABC.SP.P* | -0.678 | 0.500 | Peripherals |
| *ABC.SP.P1* | -0.796 | 0.560 | Peripherals |
| *ABC.SP.S* | -1.255 | 0.444 | Peripherals |
| *flhC* | -0.371 | 0.408 | Peripherals |
| *secA* | 0.412 | 0.620 | Peripherals |
| *secE* | 0.100 | 0.500 | Peripherals |
| *phzF* | -0.337 | 0.571 | Peripherals |
| *qseC* | -0.355 | 0.571 | Peripherals |
| *kdpE* | -0.355 | 0.571 | Peripherals |
| *lasA* | -0.107 | 0.563 | Peripherals |
| *oppF* | -0.566 | 0.444 | Peripherals |
| *crp* | -0.371 | 0.408 | Peripherals |
| *ribD* | -1.292 | 0.000 | Peripherals |
| *secDF* | -0.355 | 0.449 | Peripherals |
| *ahlD* | -1.122 | 0.000 | Peripherals |
| *rpfG* | -0.099 | 0.563 | Peripherals |
| *rpfF* | -0.099 | 0.469 | Peripherals |
| *K14645* | -0.355 | 0.612 | Peripherals |
| *oppB* | -0.371 | 0.245 | Peripherals |
| *oppC* | 0.856 | 0.496 | Peripherals |
| *trbB* | -0.678 | 0.000 | Peripherals |
| *trbF* | 0.412 | 0.580 | Peripherals |
| *trbG* | -1.292 | 0.375 | Peripherals |
| *trbI* | 1.163 | 0.486 | Peripherals |
| *g_Ralstonia* | 0.156 | 0.519 | Peripherals |
| *gmuG* | 2.201 | 0.734 | Connectors |
| *E2.5.1.54* | 0.549 | 0.720 | Connectors |
| *trpE* | 0.999 | 0.750 | Connectors |
| *ACSL* | -0.800 | 0.625 | Connectors |
| *livG* | -1.249 | 0.667 | Connectors |
| *livM* | -0.866 | 0.720 | Connectors |
| *ABC.PE.S* | 1.270 | 0.714 | Connectors |
| *ABC.SP.A* | -0.371 | 0.694 | Connectors |
| *flhD* | 0.811 | 0.667 | Connectors |
| *secB* | 0.866 | 0.765 | Connectors |
| *secG* | 0.999 | 0.656 | Connectors |
| *secY* | 0.811 | 0.764 | Connectors |
| *SRP54* | 0.923 | 0.653 | Connectors |
| *ftsY* | 2.201 | 0.657 | Connectors |
| *yajC* | 1.777 | 0.643 | Connectors |
| *yidC* | 1.729 | 0.719 | Connectors |
| *hfq* | 2.083 | 0.738 | Connectors |
| *trbL* | -0.371 | 0.694 | Connectors |
| *qseB* | 0.577 | 0.656 | Connectors |
| *glrK* | -0.866 | 0.640 | Connectors |
| *glrR* | 0.100 | 0.778 | Connectors |
| *sdiA* | 1.449 | 0.741 | Connectors |
| *lsrF* | 0.000 | 0.667 | Connectors |
| *zur* | -1.249 | 0.667 | Connectors |
| *rpfC* | -1.292 | 0.625 | Connectors |
| *oppA* | -0.107 | 0.625 | Connectors |
| *oppD* | 1.155 | 0.660 | Connectors |
| *oprM* | 0.856 | 0.711 | Connectors |
| *mexH* | 0.156 | 0.765 | Connectors |
| *expR* | -0.678 | 0.667 | Connectors |
| *fusK* | 0.577 | 0.656 | Connectors |
| *fusR* | -0.289 | 0.640 | Connectors |
